# The Role of Anthocyanin in Modulating Diabetic Cardiovascular Disease and Its Potential to Be Developed as a Nutraceutical

**DOI:** 10.3390/ph15111344

**Published:** 2022-10-30

**Authors:** Syaifuzah Sapian, Izatus Shima Taib, Haliza Katas, Jalifah Latip, Satirah Zainalabidin, Zariyantey Abd Hamid, Nur Najmi Mohamad Anuar, Siti Balkis Budin

**Affiliations:** 1 Center for Diagnostic, Therapeutic and Investigative Studies, Faculty of Health Sciences, Universiti Kebangsaan Malaysia, Kuala Lumpur 50300, Malaysia; 2 Centre for Drug Delivery Research, Faculty of Pharmacy, Universiti Kebangsaan Malaysia, Kuala Lumpur 50300, Malaysia; 3 Department of Chemical Sciences, Faculty of Science and Technology, Universiti Kebangsaan Malaysia, Bangi 46300, Malaysia; 4 Programme of Biomedical Science, Centre for Toxicology and Health Risk Research, Faculty of Health Sciences, Universiti Kebangsaan Malaysia, Kuala Lumpur 50300, Malaysia

**Keywords:** vascular dysfunction, cardiac dysfunction, cyanidin, delphinidin, oxidative stress, inflammation, fibrosis, apoptosis, diabetic cardiomyopathy

## Abstract

Cardiovascular disease (CVD) is directly linked to diabetes mellitus (DM), and its morbidity and mortality are rising at an alarming rate. Individuals with DM experience significantly worse clinical outcomes due to heart failure as a CVD consequence than non-diabetic patients. Hyperglycemia is the main culprit that triggers the activation of oxidative damage, inflammation, fibrosis, and apoptosis pathways that aggravate diabetic CVD progression. In recent years, the development of phytochemical-based nutraceutical products for diabetic treatment has risen due to their therapeutic properties. Anthocyanin, which can be found in various types of plants, has been proposed for preventing and treating various diseases, and has elicited excellent antioxidative, anti-inflammation, anti-fibrosis, and anti-apoptosis effects. In preclinical and clinical studies, plants rich in anthocyanin have been reported to attenuate diabetic CVD. Therefore, the development of anthocyanin as a nutraceutical in managing diabetic CVD is in demand. In this review, we unveil the role of anthocyanin in modulating diabetic CVD, and its potential to be developed as a nutraceutical for a therapeutic strategy in managing CVD associated with DM.

## 1. Introduction

The association between cardiovascular disease (CVD) and diabetes mellitus (DM) has been known for decades. CVD is one of the contributing factors that increase the death rate among people with diabetes. The major manifestations of diabetic CVD are coronary artery disease, peripheral artery disease, ischemic heart, stroke, diabetic cardiomyopathy (DCM), and heart failure [1]. In the last decade, the food industry has worked on identifying bioactive compounds in plants as they can bring health benefits, mainly in the therapeutic treatment of chronic diseases, due to their nutritional value. Currently, products based on enriched or concentrated mixtures of bioactive compounds from natural substances are widely commercialized, and are called nutraceuticals. De Felice [2] defined nutraceuticals as a group of products that stand between nutrition and pharmaceutical therapy. According to Food and Drug Administration (FDA) regulations, nutraceuticals have been classified as dietary supplements that come from extracts, concentrates, or a combination of vitamins, minerals, or herbs for human use to supplement the diet by increasing the total dietary intake [3]. Nutraceuticals have attracted researchers towards the further study of their potential in ameliorating diseases due to their capability to elicit health benefits, and some of the formulations have been experimentally tested [4]. Hence, the development of nutraceuticals is clinically relevant as these products exert therapeutic potential by modulating disease progression mechanisms, similar to pharmacological agents, as a treatment in diabetic CVD, for instance.

Anthocyanins are colored water-soluble bioactive compounds derived from a flavonoid group of polyphenols. Anthocyanins are the pigments that contribute to the colors, including red, purple, and blue, in fruits and vegetables. Traditionally, plants rich in anthocyanins have been widely used as dyes and food colorants, as well as medicine to treat various diseases. Indeed, anthocyanin has been widely studied for its medicinal properties. Anthocyanins possess antidiabetic [5], anticancer [6], anti-inflammatory [7,8], antimicrobial [9], and antiobesity [10] effects, as well as aiding the prevention of CVD [11]. In cardiovascular pathology, anthocyanin has been reported to exert cardioprotective effects against isoproterenol-induced myocardial infarction [12]. In addition, one study reported that anthocyanin alleviated vascular inflammation in endothelial cells [13].

Numerous studies have portrayed the benefits of anthocyanin in ameliorating diabetic CVD by targeting oxidative stress, inflammation, fibrosis, and apoptosis mechanisms [10,14,15,16]. Therefore, anthocyanins extracted from edible plants have the potential to be incorporated as pharmaceutical ingredients. However, anthocyanin exhibits low bioavailability and rapid degradation [17]. The low bioavailability of anthocyanin is caused by poor absorption in the gut due to the degradation of its structure. Hence, preserving the anthocyanin structure in nutraceutical form is vital to shield against acidic pH in the gut environment. Therefore, the development of nutraceuticals that contain anthocyanin is crucial to overcome these disadvantages. Nevertheless, anthocyanin can be found in various types of plants. Hence, using anthocyanin as a therapeutical strategy is advantageous. To the best of our knowledge, there is no review that focuses on the role of anthocyanin in modulating diabetic cardiovascular disease and its potential to be developed as a nutraceutical. Thus, this review aims to unravel the role of anthocyanin in modulating diabetic CVD by narratively reviewing the pertinent evidence in animal and human studies. We also provide insight into the kinetic and dynamic potential of anthocyanin to be developed as a nutraceutical. The search strategy for this literature review involved utilizing the PubMed database using relevant keywords, including anthocyanin, diabetes mellitus, cardiovascular disease, in vivo, in vitro, clinical study, and nutraceutical.

## 2. Diabetic Cardiovascular Disease

Diabetes mellitus (DM) is a metabolic disease associated with abnormally high blood glucose levels that is predicted to affect 693 million adults by 2045 across the world [18]. DM has evolved into one of the most deadly and evident chronic diseases of all time, resulting in life-threatening costly complications and a shortened life expectancy [19]. The number of diabetic patients keeps increasing, doubling every three decades worldwide [20]. It is ranked as the third highest risk factor and top ten cause of death globally, killing about 1.6 million people every year worldwide due to various related complications, such as CVD [21]. DM-induced CVD causes an economic burden for low- and middle-income countries, and contributes to poverty due to raised healthcare costs and high out-of-pocket expenses [22].

**Figure 1:**
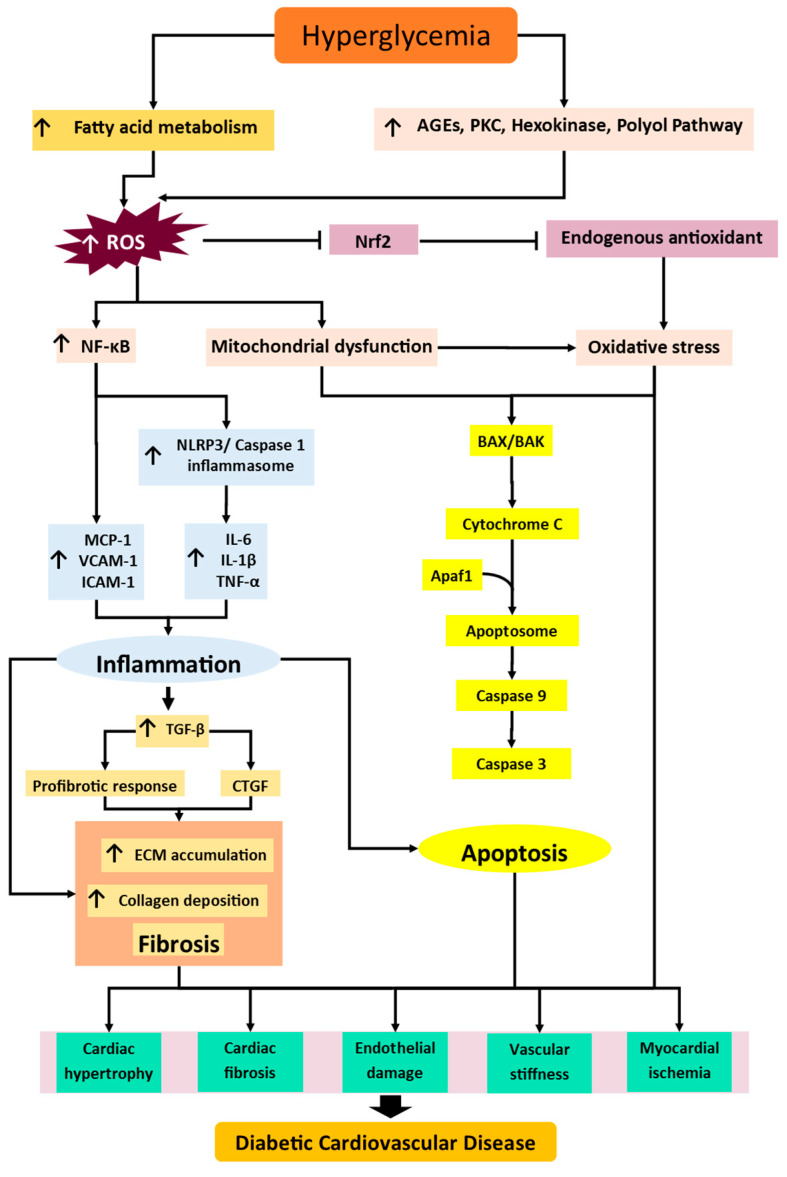
The illustrated mechanisms propose the pathogenesis of diabetic CVD. Hyperglycemia is the main culprit that induces and enhances the activation of fatty acid metabolism and nonoxidative glucose pathways, AGEs, polyol, hexosamine, and PKC pathways, which cause high generation of ROS. ROS causes downregulation of Nrf2, which reduces the activity and level of endogenous antioxidants, which ultimately causes oxidative stress. ROS also triggers the activation of NF-κB and raises the inflammasome, NLRP3, and inflammatory cytokines (TNF-α, IL-6, IL-1β, MCP-1, ICAM-1, VCAM-1), which leads to inflammation. The inflammatory response upregulates TGF-β and triggers the profibrotic response, as well as CTGF, which further causes ECM accumulation, collagen deposition, and, in the end, fibrosis. ROS causes mitochondrial dysfunction and further enhances oxidative stress. Mitochondrial dysfunction and oxidative stress triggers the cascade of apoptosis by activating apoptosomes and caspases. These mechanisms ultimately cause cardiac hypertrophy, cardiac fibrosis, endothelial dysfunction, vascular stiffness, myocardial ischemia, and ultimately, diabetic CVD. Abbreviations: AGE, advanced glycation end product; PKC, protein kinase C; ROS, reactive oxygen species; Nrf2, nuclear factor erythroid 2-related factor 2; NF-κB, nuclear factor kappa-light-chain-enhancer of activated B cells; NLRP3, NLR family pyrin domain containing 3; TNF-α, tumor necrosis factor alpha; IL, Interleukin; MCP-1, monocyte chemoattractant protein-1; ICAM-1, intercellular adhesion molecule 1; VCAM-1, vascular cell adhesion protein 1; CTGF, connective tissue growth factor; ECM, extracellular matrix; TGF-β, transforming growth factor beta ; Apaf-1, apoptotic protease activating factor 1. Arrow represent the subsequent event/product in the pathway.

CVD is the most common cause of death each year worldwide [23]. In addition, DM poses a significant risk factor for the development of CVD, which ultimately results in an increased mortality rate among diabetic patients. Hence, it is crucial to manage DM properly since the early stage of cardiovascular complications may already appear in the prediabetes stage before diagnosis [24]. The development of cardiovascular complications in diabetic conditions is aggravated by risk factors including uncontrolled hyperglycemia, dyslipidemia, hypertension, and obesity, which further elevate CVD mortality rates [25]. Hyperglycemia leads to cellular damage as glucose transport across cell membranes is not regulated normally, either because insulin is deficient or the cells have become insulin resistant; this causes high glucose concentrations and glucose metabolite bombardment, which can cause disease-related end-organ damage. High glucose levels over time play an essential role in CVD progression. The major mechanisms involved in diabetic CVD include oxidative stress (which propagates to inflammation), fibrosis, and apoptosis pathways [26,27,28,29], as illustrated in Figure 1.

Oxidative stress plays a crucial part in the pathogenesis of diabetes-induced cardiovascular complications. During the early stages of diabetes, the imbalance of glucose metabolism shifts to fatty acid metabolism to maintain sufficient levels of adenosine triphosphate in the cells since glucose utilization is depleted. However, prolonged fatty acid metabolism cannot adequately metabolize fatty acids, resulting in intracellular lipid accumulation, which leads to mitochondrial dysfunction in the heart and vascular cells. Impaired mitochondria enhance the generation of reactive oxygen species (ROS) [30]. Furthermore, impaired glucose metabolism will also induce the activation of nonoxidative glucose pathways, including polyol, protein kinase C (PKC), hexosamine, and advanced glycation end product (AGE) pathways [31,32,33]. These pathways further incite their own ROS generation, thereby aggravating cardiovascular dysfunction [34]. Persistent increases in ROS generation, and its accumulation in the cells, hampers endogenous antioxidant defense regulated by nuclear factor erythroid 2-related factor 2 (Nrf2), leading to diabetes-associated inflammation, fibrosis, and apoptosis in CVD.

The elevation of ROS accumulation is closely linked to the inflammatory response that aggravates diabetic CVD [27,35]. The highly inflammatory environments occur due to the rise in the expression, and thus level, of proinflammatory cytokines, including tumor necrosis factor α (TNF-α), interleukin (IL)-1β, IL-6, IL-8, monocyte chemotactic protein 1 (MCP-1), intracellular adhesion molecule 1 (ICAM-1), and vascular adhesion molecule 1 (VCAM-1) [36], in the cardiovascular system. All of these cytokines are regulated by the inflammatory transcriptional regulator nuclear factor-kappa B (NF-κB) [37,38]. The influx of infiltrating inflammatory cells activated by cytokines has emerged as a significant contributor to myocardial and vascular injury development. The proinflammatory state is thought to inaugurate the extension of inflammatory mechanisms by triggering NLR family pyrin domain containing 3 (NLRP3) inflammasomes in the cardiovascular system [39]. NLRP3 inflammasome activation prompts the recruitment of pro-caspase-1 to facilitate caspase-1 activation, and the cleavage of IL-1β and IL-18 precursors to generate their active products. As a result, a plethora of inflammatory mechanisms are enhanced and favor diabetes-induced fibrosis in the heart and vasculature system [26].

Fibrosis is the initial characteristic of cardiomyopathy and vasculopathy, and is already present in the diabetic condition in the initial stage. Under diabetic conditions, the surge in ROS production, as well as AGEs, activates and increases NF-κB via phosphorylation of IkB. Activation of NF-κB induces transforming growth factor beta (TGF-β1) activation, which phosphorylates MAPK and P13K for increased production of extracellular matrix (ECM) proteins, such as collagen I, collagen III, fibrin, and laminin. TGF-β1 also induces the activation of connective tissue growth factor (CTGF) to enhance ECM production. In this condition, an overproduction of ECM proteins leads to myocardial and vascular stiffness and consequent cardiac and vascular dysfunction, ultimately resulting in cardiovascular complications.

Impaired glucose utilization alters homeostatic pathways of oxidative stress, inflammation, and fibrosis, which have been implicated as drivers in the apoptosis pathways of CVD. The proteolytic cascade of apoptosis is induced by activated caspases, which have been widely expressed in damaged cardiomyocytes and endothelial cells [40]. There are two major mechanisms in the apoptosis cascade: intrinsic and extrinsic pathways [41]. The extrinsic apoptotic pathway involves multiple death ligands, such as TNF-α and Fas, binding to their homologous receptors to trigger cell death. After that, caspase-8 and caspase-9 are activated, which then promotes the activation of executioner caspases (caspase-3, -6, and -7), leading to the eventual induction of apoptosis. The intrinsic pathway occurs in mitochondria, where the complex permeability transition pores form at the mitochondrial membrane. Cytochrome C and other mitochondrial proteins are released into the cytosol. Subsequently, cytochrome C and apoptosis protease activating factor-1 (Apaf-1) merge to generate an apoptosome, which plays a crucial role in the cleavage and upregulation of following caspases that ultimately cause cell death [42]. After taking all this into consideration, finding an alternative therapy aimed at the main contributing mechanisms involved in the development of diabetic CVD is crucial.

## 3. Anthocyanin

**Figure 2:**
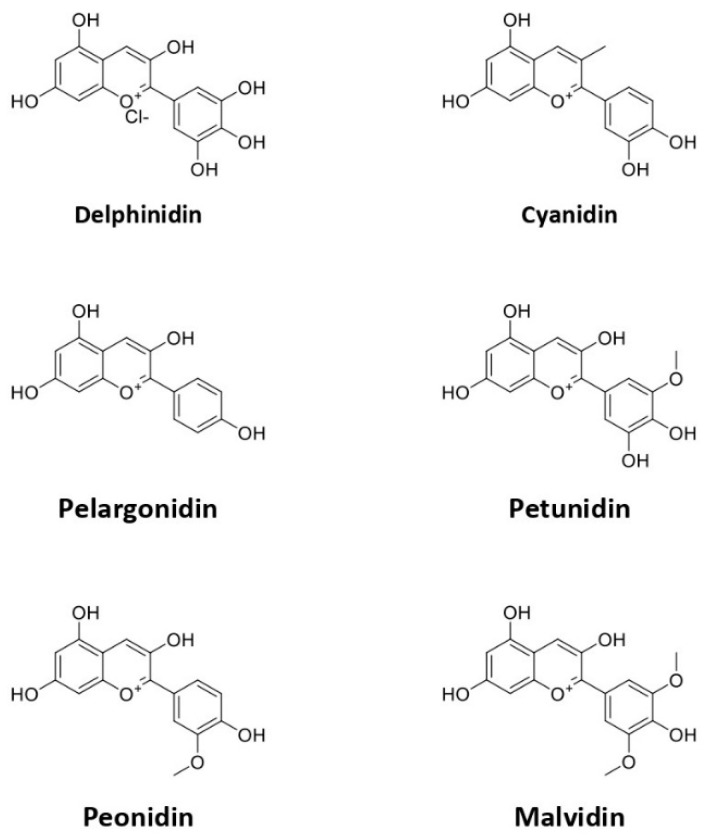
The chemical structure of subtypes of anthocyanin.

Anthocyanin is a water-soluble flavonoid group that renders various colors of purple, red, pink, and blue in plants. Anthocyanins can be found in a wide range of plants, such as roselle, blueberry, purple cabbage, and plum [43,44,45]. Anthocyanins are very sensitive to temperature, pH, and light [46]. Anthocyanin usually appears as a red pigment in acidic and blue in alkaline conditions. Furthermore, some studies reported that anthocyanin has no negative effect, even after the consumption of high doses [17,47]. The basic structure of anthocyanin is 2-phenylchromenylium (flavylium) with a combination of glucose, galactose, and rhamnose. However, anthocyanins can form various types, including pelargonidin, delphinidin, petunidin, cyanidin, and malvidin (Figure 2), all of which are differentiated based on the B-ring on the basic structure [48].

### Bioavailability of Anthocyanin

The bioavailability of anthocyanin refers to the anthocyanin content that is absorbed, circulated, metabolized, and distributed to targeted tissue [49]. Anthocyanin has poor bioavailability, with only 1 to 2% of consumed anthocyanin present within plasma. While anthocyanin is quickly absorbed, it is rapidly metabolized and excreted. [50,51]. The low bioavailability of anthocyanin is related to their stability, lack of site-specificity in distribution, quick excretion and clearance from the system, and interactions with food matrices [52]. In the gut, anthocyanins are absorbed, before traveling through the portal vein to the liver. They are metabolized, secreted, and reabsorbed in the liver. Anthocyanins were detected in their original form in plasma within 6 min after oral administration in an in vivo investigation, which showed that they are initially absorbed in the stomach by a bilitranslocase-mediated mechanism. They can, however, be discovered in bile within 20 min as methylated and conjugated derivatives, indicating that anthocyanin metabolites are quickly produced in the liver and removed through bile [53].

Food digestion is a pH-dependent process. Since anthocyanin is very sensitive to pH, it is aided in transformation by enzymes in the small intestine via hydrolyzation [54]. Additionally, anthocyanins are quickly assimilated in the small intestine of rats, and can be identified in their original form and methylated derivatives in circulation within 25 min. However, they are also eliminated into the bile and urine as intact glycosides or methylated/glucuronidated derivatives [55]. In the small intestine, anthocyanins can be absorbed through deglycosylation to aglycones, accompanied by passive diffusion via intestinal epithelium or by an active transport process involving bilitranslocase or the intestinal sodium-dependent glucose transporter 1 (SGLT1) [56]. Anthocyanins are converted into low-molecular-weight catabolites once they enter the large intestine, where they can either be expelled in the feces in 2 to 8 h or be reabsorbed. The majority of anthocyanins in the blood are present as their metabolites, which are less pharmacologically active than the original molecules. Anthocyanin’s prospective applications in the food and pharmaceutical industries are constrained by its instability, high susceptibility to environmental factors, and low extraction capability [57,58]. Nevertheless, anthocyanin can be found abundantly in plants, and has been proven to exert therapeutic effects in alleviating various diseases, particularly diabetic CVD. Hence, the development of anthocyanin as a nutraceutical is critical.

## 4. The Role of Anthocyanin in Modulating Mechanisms of Diabetic Cardiovascular Disease

**Figure 3:**
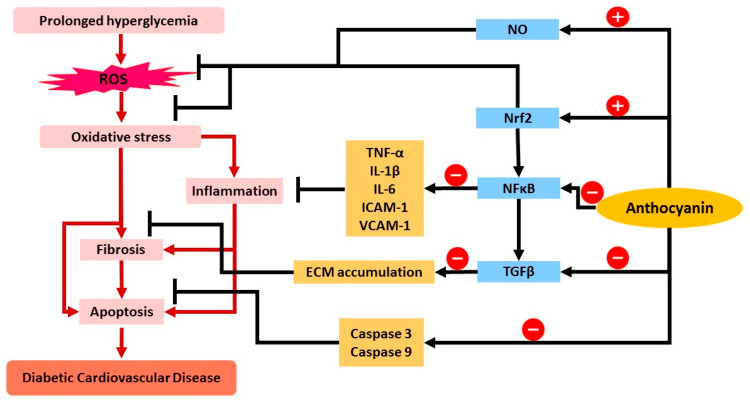
The summary of the role of anthocyanin in attenuating mechanisms of diabetic CVD. Anthocyanin increases NO bioactivity and upregulation of Nrf2, which induces the production of endogenous antioxidants and limits oxidative stress. Anthocyanin also has been proposed to downregulate NF-κB and lead to reduced expression and production of cytokines involved (TNF-α, IL-1β, IL-6, ICAM-1, VCAM-1) and further reduces the inflammatory response. NF-κB downregulates TGF-β expression as well as anthocyanin treatment, and causes reduced ECM accumulation and limits fibrosis. Anthocyanin also reduces caspase-3 and caspase-9 levels, which further attenuates apoptosis. Abbreviations: ROS, reactive oxygen species; NO, nitric oxide; Nrf2, nuclear factor erythroid 2-related factor 2; NF-κB, nuclear factor kappa-light-chain-enhancer of activated B cells; TGF-β, transforming growth factor beta; TNF-α, tumor necrosis factor alpha; IL, interleukin; ICAM, intercellular adhesion molecule 1; VCAM, vascular cell adhesion protein 1; ECM, extracellular matrix. (-) sign indicates the inhibition/downregulation of related gene/protein. Black arrow indicates the activity of anthocyanin while red arrow indicates the subsequent event/product in the pathway.

Anthocyanins have been at the center of attention over the past two decades, particularly due to the protective effects against CVD when consuming anthocyanin-rich food. Accumulating evidence suggests that anthocyanin treatment is able to improve dyslipidemia [59], promote vascular protection [60], ameliorate atherosclerosis [61], counteract obesity [62], and attenuate DCM [63]. Many studies acknowledge the potential of anthocyanin in modulating diabetic cardiovascular disease by modulating oxidative damage, inflammation, fibrosis, and apoptosis, as summarized in Table 1. Meanwhile, Figure 3 illustrates the potential mechanisms exerted by anthocyanin in modulating CVD in diabetic conditions.

### 4.1. Antioxidative

Anthocyanin has been studied widely due to its antioxidant activity. In a previous study, anthocyanin-rich tart cherry extract was able to prevent the decrease in water-soluble antioxidant capacity and increased superoxide dismutase (SOD) activity in type 2 DM (T2DM) models [10]. Furthermore, Huang and his colleagues [14] have demonstrated that administrations of blueberry anthocyanin extract, malvidin, malvidin-3-glucoside, and malvidin-3-galactoside were able to significantly ameliorate injured endothelial cells, induced by high glucose level, by elevating endogenous SOD and heme oxygenase-1 (HO-1), lowering ROS production, and downregulating NADPH oxidase isoform 4 (NOX4) expression. The same study also showed that anthocyanin effectively induced vasodilatory effects by increasing nitric oxide (NO) and its promoter, endothelial NO synthase (eNOS), and peroxisome proliferator-activated receptor-γ (PPARγ) levels, decreasing the vasoconstrictor angiotensin-converting enzyme (ACE), xanthine oxidase-1 (XO-1), and low-density lipoprotein (LDL) levels, as well as via the activation of the phosphoinositide 3-kinase (PI3K)/Akt signaling pathway and the breakdown of the PKCζ pathway.

In addition, cyanidin 3-glucoside, an anthocyanin, has been reported to exhibit antioxidative properties by elevating SOD activity and diminishing malondialdehyde (MDA) content in diabetic hearts [70]. A recent study demonstrated that anthocyanin derived from berries exerted cytoprotective effects against H_2_O_2_-induced oxidative stress in diabetic aortic endothelial cells by attenuating cytotoxicity induced by H_2_O_2_ [71]. Interestingly, anthocyanin has been reported to significantly reduce the levels of ROS-generating enzymes, NOX2 and NOX4, indicating that anthocyanin contributes to limiting oxidative stress in diabetic vasculature [67]. In an in vitro study, anthocyanin-rich sour cherry extract exhibited potent radical scavenging capacity by significantly reducing ROS in diabetic human umbilical vein endothelial cells (HUVEC) cells compared to under normoglycemia conditions [5].

The beneficial effects of anthocyanin in ameliorating insulin resistance in endothelial cells have been well documented. An earlier study manifested that cyanidin-3-O glucoside was able to elicit the response in modulating the transcription factor Nrf2, which is crucial in the production of endogenous antioxidants; this in turn countered the decrease in NO levels and restored the expression of atherothrombotic factors ET-1 and plasminogen activator inhibitor-1 (PAI-1) [68,72]. The same study also indicated that anthocyanin pretreatment was able to limit IRS-1 serine phosphorylation and elevate tyrosine phosphorylation, as well as restore P13K/Akt axis signaling altered by insulin resistance, increase Akt and eNOS phosphorylation, and restore inhibitory kappa-B kinase β (IKKβ) and Jun-N terminal kinase (JNK); these protective effects occurred via the activation of Nrf2. In a streptozotocin-induced diabetes model, cyanidin 3-glucoside was confirmed to attenuate the increase in MDA content and reduce the activity of SOD in the vascular system [64]. This study indicates that anthocyanin has the potential to be developed as a nutraceutical in targeting CVD related to DM by the modulation of oxidative stress mechanisms.

### 4.2. Anti-Inflammation

A body of growing evidence has revealed that anthocyanin is able to exert anti-inflammatory properties in ameliorating diabetic CVD. The anthocyanin compound has been proven to inhibit the elevation of IL-17, IL-1β, TNF-α, and IL-6 in DCM models, suggesting that anthocyanin has anti-inflammatory effects [63,70]. Another study has elaborated the same finding but using a different model, that of diabetic endothelial cells, and reported that anthocyanin successfully suppressed the gene expression level of inflammatory cytokines, including IL-6, IL-8, and IL-1α. On top of that, the expression profiles of inflammation marker proteins, including cyclooxygenase 2 (COX-2), toll-like receptor 4 (TLR4), p-IKKα/β, p-IκBα, IL-6, and p-NF-κB, in diabetic hearts have been mitigated with anthocyanin treatment [66]. Another study suggested that anthocyanin from berries limits the inflammatory response in aortic endothelial cells under hyperglycemic conditions by reducing the expression of inflammatory cytokines, including IL-6 and IL-1β, via the inactivation of the NF-κB signaling pathway [71].

The influence of strawberries, rich in anthocyanin, on endothelial inflammation of diabetic vasculature was well postulated in a previous study conducted by Petersen and colleagues [69]. This study revealed the effect of strawberry supplementation in regulating the inflammatory markers both in protein and gene expression by suppressing MCP-1/JE, KC, ICAM1, and VCAM1, and reducing the expression of IKKβ and IκBα in the aortic vessel of the diabetic model. Hence, this finding suggests that anthocyanin has a promising effect in ameliorating atherosclerosis, since monocytes binding to the aortic vessel, followed by their transmigration into subendothelial space, plays a major role in atherosclerosis development through the attenuation of NF-κB signaling. Moreover, anthocyanin from blackcurrant has been confirmed to elicit an anti-inflammatory response in diabetic vasculature by markedly reducing the protein levels of inflammatory cytokines, IL-6, TNF-α, and IL-1β [16].

### 4.3. Anti-Fibrosis

One of the mechanisms that worsen the progression of diabetic CVD is fibrosis. Nevertheless, few studies have discovered the role of anthocyanin in hindering fibrosis. A past study that used purple rice anthocyanin extract showed that cardiac fibrosis in hyperglycemic conditions was successfully attenuated by decreasing the activation of metalloproteinase (MMP)-9 and reversing TIMP-1 protein expression [66]. Furthermore, the same study also proved that anthocyanin treatment reduced the levels of FGF2, phosphorylated extracellular regulated signal kinase (p-ERK1/2), uPA, as well as downregulated the protein expression of TGF-β, p-MEK1/2, and CTGF. Another study conducted by Yue and colleagues [63] confirmed that anthocyanin could attenuate inflammation in diabetic models by diminishing the accumulation and protein levels of collagen I and III in the cardiomyocytes.

Blackcurrant is a fruit with high anthocyanin contents, including delphinidin-3-O-glucoside, delphinidin-3-O-rutinoside, cyanidin-3-O-glucoside, and cyanidin-3-O-rutinoside [16]. Blackcurrant was found to modulate cardiac fibrosis and hypertrophy by the diminution of cardiac remodeling marker expression levels, TGF-β1, and collagen IV in the left ventricle of hearts under high glucose concentration [16]. Additionally, the same study also indicated that blackcurrant treatments can reduce the elastin fibers in the thoracic aorta, which highlights the role of anthocyanin in modulating fibrosis pathways.

### 4.4. Anti-Apoptosis

Apoptosis plays a crucial part in diabetic CVD etiologies. However, anthocyanin is a well-known bioactive compound in ameliorating DM that stimulates apoptosis in CVD. This has been confirmed by a study that revealed that anthocyanin treatment resulted in a higher-level response of anti-apoptotic Bcl-2 and a lower-level response of proapoptotic caspase-3 and BAX [70]. Moreover, anthocyanin that contains high levels of cyanidin-3-glucoside efficiently protects hearts in DM condition from apoptosis by reducing cytochrome c accumulation, suppressing protein levels of Fas ligand, Fas receptor, Fas-associated death domain (FADD), and activating caspase-8 [67]. This study also discovered that the protein levels of proapoptotic proteins, Bad, Bak, and caspase-9, were ameliorated with anthocyanin treatment. Additional to being a regulator of apoptosis that induces endoplasmic reticulum stress, CHOP has been successfully downregulated by the treatment of anthocyanin in diabetic conditions [65]. The study of anthocyanin in alleviating diabetic CVD via oxidative stress, inflammation, fibrosis, and apoptosis should be further explored in future to provide more information regarding the therapeutic potential of anthocyanin in alleviating diseases, especially diabetic CVD.

## 5. Anthocyanin in Clinical Studies for Cardiovascular Disease

Anthocyanin has been explored in the clinical stage, particularly in CVD. Some human trials have assessed CVD-related risk factors after anthocyanin-containing interventions, and have shown that anthocyanin exhibits beneficial effects [73]. A clinical study proved that anthocyanin supplementation improved cholesterol efflux capacity in subjects with dyslipidemia after consuming 80–320 mg/day of anthocyanin for 12 weeks [74]. Another study evaluated the efficacy of anthocyanin-Maqui berry extract (Delphinol) on overweight subjects, and found that anthocyanin possesses a nutritional influence on lipid peroxidation and reduced ox-LDL levels, suggesting the protective effects of anthocyanin against CVD [75]. Furthermore, hypercholesterolemic individuals who were treated with 320 mg of purified anthocyanins for 24 weeks demonstrated reduced platelet-derived chemokines and restored lipid profiles, indicating that anthocyanin is a potential candidate in the prevention of atherosclerosis [76].

Moreover, subjects with postprandial metabolic imbalances that consumed anthocyanin-rich Queen Garnet Plum as an intervention were reported to have reduced levels of inflammatory markers, i.e., C-reactive protein, accompanied by lower concentrations of vascular IL-6, which indicates that anthocyanin may reduce the cardiovascular risk associated with endothelial dysfunction and inflammatory responses [77]. Anthocyanin supplementation of 320 mg twice daily for 28 days alleviated thrombogenic progression by reducing monocyte–platelet aggregate formation and platelet aggregation induced by adenosine diphosphate, collagen, and arachidonic acid in overweight or obese populations [78]. Recently, a study was conducted aiming to observe the effects of cranberry juice, which has high anthocyanin contents, on cardiovascular risk factors in adults with elevated blood pressure. Overweight individuals exhibited modest effects in terms of 24 h ambulatory and diastolic blood pressure and lipoprotein profile [79].

As we discussed above, anthocyanin has been well documented regarding its role in alleviating CVD-related risk factors. Therefore, anthocyanin can exert its effects as a therapeutic intervention in ameliorating DM-induced CVD (Table 2). Several clinical approaches were conducted that aimed to explain the role of anthocyanin in preventing CVD associated with DM. In a double-blind, parallel-arm, randomized controlled trial, 52 men with T2DM supplemented with 22 g freeze-dried blueberries rich in anthocyanins for 8 weeks exhibited reduced concentrations of serum triglyceride, HbA1c, and fructosamine, suggesting that anthocyanins may beneficially affect cardiometabolic health [80]. Another randomized and controlled trial was conducted on men and women of midlife with prediabetes or early untreated diabetes to investigate the effects of purified anthocyanins on CVD associated with DM [81]. They found that the 12-week consumption of purified anthocyanins had favorable effects in terms of reducing HbA1c, LDL cholesterol, and apolipoprotein B, and enhancing apolipoprotein A1, which highlights that anthocyanins have beneficial effects on glycemic control and lipid regulation among patients with cardiometabolic disorders.

Another clinical double-blind, placebo-controlled, parallel study was carried out using anthocyanin-rich blueberries as the intervention for patients with metabolic syndrome (hyperglycemia, high blood pressure, central adiposity, high triglyceride level, and diminished levels of high-density lipoprotein (HDL)) for 6 months [82]. At the end of the study, they found that a daily intake of one cup of anthocyanin-rich blueberries boosted endothelial function, improved arterial stiffness, and enhanced HDL cholesterol and apolipoprotein A1 levels, which was believed to be due to the recovery of vascular and lipid status. However, insulin resistance and peripheral, hepatic, and adipose tissue insulin sensitivity were not affected by the study’s anthocyanin-rich blueberry.

Continuing with another study performed with bilberry extract supplementation that was rich in anthocyanin content, using a randomized, double-blind, placebo-controlled, and crossover approach, subjects with T2DM that ingested 1.4 g/day of bilberry extract for 4 weeks exhibited decreased HbA1c values; however, there was no significant difference in lipid status, antioxidant status, oxidative stress, or anti-inflammatory status [83]. Furthermore, in a quasi-experimental study, 19 diabetic women were given concentrated sour cherry juice, which contains mainly anthocyanin, daily for 6 weeks, and it was shown that its consumption was able to reduce blood pressure and HbA1c, as well as total cholesterol and LDL cholesterol [84].

Due to the preclinical effects of anthocyanins, many human studies have been performed over the past decade to discover the benefits of anthocyanins observed in vitro, and in vivo studies have been applied to diabetic patients with cardiometabolic syndrome. However, most clinical trials use a variety of dosages and different sources of anthocyanins. The dosages used are either too small or too high for the optimal effects of anthocyanins. Moreover, most of the clinical studies are frequently conducted on participants that have CVD risk factors, especially diabetic patients. Nonetheless, the studies presented did not show reduced rates of diabetic CVD. Not only this, but the limited bioavailability of anthocyanins also makes it difficult to interpret the cause of neutral results of anthocyanin intervention, either due to dosage variabilities, intervention duration, anthocyanin sources, lack of potency of natural anthocyanins, or ineffectiveness of the anthocyanin in treating human diabetic CVD regardless of potency and dose. Furthermore, the number of clinical studies that use purified anthocyanin is limited. Many studies used different sources of anthocyanin and various extract types, which means it contains other types of bioactive compounds. Thus, it is enigmatic to know whether the beneficial effects of CVD related to DM are solely because of anthocyanin itself or synergistic effects between anthocyanin and other compounds found in variable sources of anthocyanins. Nevertheless, anthocyanin may have the potential to alleviate DM-induced CVD. Hence, the incorporation of anthocyanin in nutraceutical form is much needed to increase its stability and bioavailability, and to be tested in the clinical trial stage.

**Table 2:** Summary of effects of anthocyanin in in clinical studies of diabetic cardiovascular disease.

Anthocyanin Form and Sources	Subject	Study Design	Dose and Duration	Outcomes	Reference
Sour cherry juice	19 diabetic women	Quasi-experimental study	40 g/day for 6 weeks	Reduced blood pressure and HbA1c level, decreased total cholesterol and LDL cholesterol level	Ataie-Jafari et al., 2008 [84]
Purified anthocyanins	160 participants with prediabetes	Double-blind, randomized, placebo-controlled trial	320 mg/day for 12 weeks	Reduced HbA1c, LDL cholesterol, apolipoprotein B, and enhanced apolipoprotein A1	Yang et al., 2017 [81]
Freeze-dried blueberry	115 with metabolic syndrome	Double-blind, parallel-arm, randomized controlled trial	13 g and 26 g for 6 months	Improved endothelial function and systemic arterial stiffness, enhanced HDL cholesterol levels and increased apolipoprotein A1	Curtis et al., 2019 [82]
Freeze-dried blueberry	52 men with T2DM	Double-blind, parallel-arm, randomized controlled trial	22 g for 8 weeks	Reduced serum triglyceride level, HbA1c, and fructosamine	Stote et al., 2020 [80]
Bilberry extract	20 patients of T2DM	Randomized, double-blind, placebo-controlled crossover study	1.4 g/day for 4 weeks	Reduced HbA1c level	Chan et al., 2021 [83]

## 6. Development of Anthocyanin as a Nutraceutical

Nutraceuticals are defined as any products that are derived from food sources that provide medical or health benefits, including the prevention and treatment of disease. Nutraceuticals are dietary medicines that provide a concentrated dose of an alleged bioactive ingredient, proven to be nontoxic to humans, from a food, delivered in the non-food matrix, and used to improve health in dosage a form more significant than those derived from typical foods. In addition, nutraceuticals could be a powerful tool to be used beyond diet and before drugs to prevent and treat pathological conditions, for example, in subjects who may not be eligible for conventional pharmaceutical therapy [85].

The development of anthocyanin as a nutraceutical has gained tremendous interest in recent years. It is admirable that interest has been resurgent over the last 20 years, driven by the food industry, consumers, and the scientific community. Consumers prefer natural compounds because they have fewer side effects than synthetic ingredients. During this time, industries have overcome numerous storage and stability-related challenges, and are now thinking about bringing the best anthocyanin products from research projects to market. The structure of anthocyanin, regarding the type, number, and position of substituent groups, such as hydroxyl and methyl groups, plays a role that contributes to its instability. In addition, anthocyanin is susceptible to environmental factors, such as light, heat, pH, and oxygen [86]. The degradation of anthocyanin and its color fading affects the quality of food-rich anthocyanin and reduces its bioactivity [57]. Despite this, anthocyanin has been proven to exert cardioprotective effects against CVD, particularly diabetic CVD. Hence, it is vital to preserve the anthocyanin structure and enhance its stability and bioavailability in the nutraceutical form. Due to the variety of studies conducted involving anthocyanins, it is time to translate this research into products for human use, particular with emphasis on CVD associated with DM parameters.

The dose of the anthocyanin from the typical food sources can be variable, and thus not fix. Hence, the amount of anthocyanin needed to exert therapeutic effects is commonly unpredictable since the amount of anthocyanin in food sources differs between sources. Thus, the development of anthocyanin nutraceutical is crucial for dosage uniformity in order to achieve beneficial effects in alleviating diseases. The nutraceutical efficacy depends on the bioavailability, absorption, stability, and the excipient used in the nutraceutical development process. The dosage determination in nutraceutical development for anthocyanin is depends on the effective dose used from the food sources or extracts based on past studies [87]. However, in some cases, the dose of the nutraceutical can also be influenced by the excipient used as the drug delivery system. As an example, the incorporation of anthocyanin into nanoparticles, and therefore on a nanoscale, which can enhance its bioavailability. Hence, a lower dose is needed to provide significant effects compared to free anthocyanin.

Chemical alterations and novel drug design techniques such as nanotechnology are highly desired to increase the bioavailability and stability of anthocyanins for clinical use [88,89]. Entrapped bioactive compounds in the encapsulated forms nearly exclusively provide protection and controlled release of the compounds [53]. Increasing the bioavailability and improving the distribution of anthocyanins is possible through new research avenues, such as nanoencapsulation [90]. Nanoencapsulation has several benefits, including flavor preservation, improved thermal and oxidative stability of bioactive substances, overcoming the drawbacks of high volatility, managing substance release, and increased bioavailability. According to a previous study, anthocyanin that has been nanoencapsulated may tolerate a wider pH range, the presence of metal ions, and an increase in temperature, while still being able to neutralize free radicals [91]. In an earlier study, anthocyanin-loaded β-lactoglobulin nanoparticles were stable against pH in the gastrointestinal system, showing a higher retention rate than the unencapsulated one [92].

The capacity of anthocyanins to noncovalently interact with macromolecules to create stable nanostructures is one of their unique chemical traits. [93]. Hence, the application of nanocarrier systems such as natural polymers, polysaccharides, proteins, metals, and lipids, or the combination of carriers, can improve encapsulation efficiency and functionality [94,95,96]. They are highlighted as being particularly promising for usage as a wall material because they exhibit great biodegradability and biocompatibility and have numerous natural sources of extraction [97]. Additionally, excipients such as chitosan, hydrochloride, inulin, and carboxymethyl chitosan utilized to construct nanocomplexes with anthocyanins as carriers demonstrated maximum anthocyanin retention rate, reduced particle size, and excellent encapsulation efficiency. A study conducted on the formulation anthocyanin-loaded nanocomplexes prepared with chitosan hydrochloride and carboxymethyl chitosan has shown how to stabilize anthocyanin against degradation caused by storage temperature, varying pH, and white fluorescence light, which has excellent potential to be applied in the formulation of nutraceutical anthocyanin [94].

Polysaccharide-based nanostructures can shield and release the encapsulated substances into the environment and on particular physiological stimuli. Numerous benefits for anthocyanin encapsulation are provided by polysaccharides due to their physical, chemical, and functional characteristics. The creation of nanocarriers is appropriate due to the intricacy of polysaccharide structures. With this aim, encapsulated bioactive chemicals, including anthocyanins, are frequently protected and controlled during release using polysaccharides such as chitosan, cellulose and derivatives, and pectin [98]. Polysaccharide-based nanoparticles are intended to protect the encapsulated substance from the gut environment, improve responsive delivery that depends on pH, and transport the substance directly to the human intestine. Controlled intestinal re-release of anthocyanin-containing nanostructures may facilitate absorption, particularly in the integral form [91,99]. A study on formulating anthocyanin-loaded nanocomplexes prepared with chitosan hydrochloride and carboxymethyl chitosan has shown how to stabilize anthocyanin against degradation caused by storage temperature, varying pH, and white fluorescence light, which has excellent potential to be applied in the formulation of nutraceutical anthocyanin [94].

Anthocyanin is hydrophilic due to its chemical structure, and cannot penetrate the lipid bilayer of the plasma membrane by passive transport. Therefore, anthocyanin require a hydrophilic carrier. Lipids are regarded as excellent nanocarriers for the encapsulation of anthocyanin, as they can across the plasma membrane. High encapsulation efficiency, controlled release in the gut, low toxicity, and high potential for industrial production are all benefits of lipid-based nanoencapsulation. Bilayer structures (often spherical) with particular polar lipids dispersed in aqueous phases can generate lipid-based nanocapsules [100]. Lipid-based carriers, comprising solid lipid nanoparticles, nanoliposomes, and nanoemulsions, are a novel type of encapsulation system called nanostructured lipid carriers. Liposomal micelles for anthocyanin encapsulation can increase the bioavailability of anthocyanins as it is lipid-soluble and can cross the plasma membrane for a higher absorption rate. Many studies use lipid-based formulations, including anthocyanin-rich black-carrot-extract-loaded liposomes [101], anthocyanin-loaded liposomes prepared by the pH gradient loading method [102], anthocyanin-loaded liposomes using an improved supercritical carbon dioxide method [103], and nano-liposomal system based on lyophilization for encapsulating anthocyanin-rich extract of red cabbage [104]. All of these formulations have been successfully demonstrated to increase anthocyanin storage, preserve the radical scavenging activity of anthocyanin, and protect against anthocyanin degradation from pH variability, temperature, oxygen, and light.

**Figure 4:**
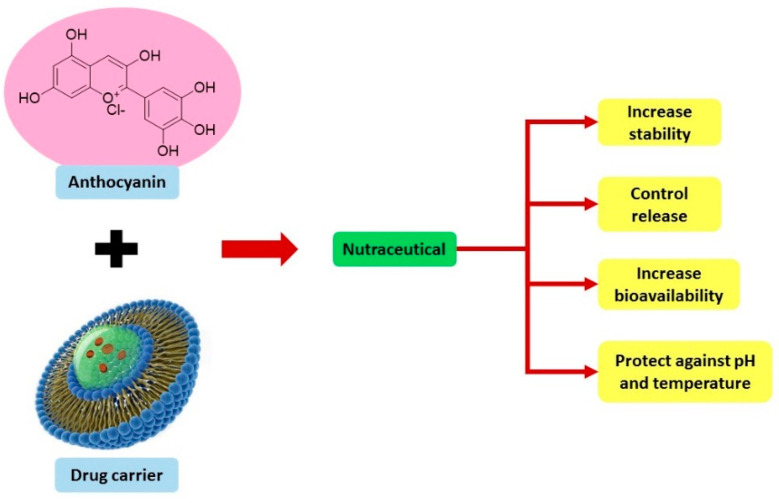
Advantage of anthocyanin in nutraceutical forms.

Research into the formulation of nutraceuticals in solid dosage form (tablet) is ongoing. Tablet formulation has advantages in that dosage and volume of the active ingredient can be managed precisely, packaged nicely, swallowed easily, and practically transported and stored easily [105]. Recently, a study formulated liposomal freeze-dried anthocyanin-enriched *Vaccinium arctostaphylos* L. fruit extract incorporated into fast-dissolving oral films that may improve patients’ compliance and effective antioxidant activity [106]. These characteristics increase the possibility of anthocyanin application as pharmacotherapy, particularly in diabetic CVD. Figure 4 summarizes the advantages of anthocyanin in nutraceutical form.

## 7. Application of Anthocyanin Nutraceutical in Diabetes Mellitus and Cardiovascular Disease

Several studies used anthocyanin nutraceuticals to determine its effect in modulating diabetic conditions and CVD. An earlier study conducted by Gharib and colleagues portrayed the treatment of diabetic animal models by anthocyanin, mainly delphinidin and cyanidin hydrochloride, in liposomal forms, and was able to decrease HbA1c glycation and provide better effects compared to free anthocyanins [107]. Additionally, anthocyanin-rich purple-sweet-potato-extract-loaded carboxymethyl chitosan alginate nanocapsules were shown to reduce the blood glucose levels of diabetic mice with 4.4 times greater efficiency compared to extract without encapsulation [108]. Anthocyanin in eggplant fruit peel extract in the form of lyophilized tablets was shown to be a promising nutraceutical in lowering hyperglycemia in diabetic rats [109]. In another study, *Bauhinia variegata* flower extract rich in anthocyanin that was incorporated in silver nanoparticles exerted antioxidant properties, and also efficiently inhibited α-amylase activity compared to extract without silver nanoparticles [110].

Moreover, dietary supplementation of encapsulated anthocyanin-loaded chitosan nanoparticles attenuated hyperlipidemic aberration in experimental rats induced by high-fat and alcohol diets by inhibiting lipid peroxidation, increasing antioxidant enzyme activity, and suppressing the development of lipogenesis [99]. Another study, suggesting the use of *Vaccinium arctostaphylos* L., reported that fruit extract that contained high anthocyanin concentrations loaded into zinc oxide nanoparticles significantly decreased fasting blood glucose and LDL and increased HDL levels in alloxan-induced diabetic models and exhibited better results compared to treatment with extract alone [111]. Recently, cardiac dysfunction caused by fibrosis was successfully attenuated by anthocyanin incorporated into hydrogel nanoparticles by maintaining glycogen content in the heart tissue and reducing MDA and hydroxyproline content in animal models. This suggests that the delivery of anthocyanin via nanoparticles may augment its retention in the body, as the capsules may shield anthocyanin from immunoglobulins and cellular components of the immune system [112].

Another study performed nanoencapsulation using low-molecular-weight chitosan from black carrot anthocyanin, and the product was shown to possess antioxidant properties in animals fed a high-fat diet [113]. In obese mice, anthocyanin-loaded niosomes ameliorated insulin resistance and glucose intolerance by reducing plasma insulin, glucose level, leptin, and total cholesterol, indicating the beneficial effects on the reversal of metabolic abnormalities associated with obesity [114]. Previously, a study proved that tablets from purple sweet potato extract rich in anthocyanins are safe to consume and exert beneficial effects, including restoring the lipid profile to normal levels and oxidative stress mitigation in rats [115].

## 8. Conclusions

Prolonged hyperglycemia is the main culprit that induces the development of diabetic CVD by activating oxidative stress, inflammation, fibrosis, and apoptosis pathways. There is growing evidence showing that anthocyanin, a flavonoid, can protect against the progression of diabetic CVD in preclinical and clinical studies by exerting its effect as antioxidative, anti-inflammation, anti-fibrosis, and anti-apoptosis component. However, studies focusing on using anthocyanin in diabetic CVD are still limited, and the sources of anthocyanin used in previous studies are variable, and it is difficult to interpret whether the effects are mainly from anthocyanin itself or synergistic effects of anthocyanin with other compounds. Furthermore, the properties of anthocyanin, including low bioavailability and poor stability, can interfere with the biological advantages of anthocyanin. Hence, the development of anthocyanin as a nutraceutical is crucial. The formulation of anthocyanin as a nutraceutical can stabilize anthocyanin structure, increase bioavailability, and enhance its shelf life, which may increase the potency of anthocyanin as a therapeutic in various diseases, particularly diabetic CVD.

## Figures and Tables

**Table 1:** Summary of effects of anthocyanin and the targeted pathways involved in diabetic cardiovascular disease.

Sources	Effects	Study Design	Dose	Duration	Pathways Involved	Reference
Commercial cyanidin-3-glucoside	Antioxidative	In vivo (T1DM)	10 mg/kg	8 weeks	Decreased MDA content and elevated SOD activity	Nasri et al., 2011 [64]
Saskatoon berry powder	Anti-apoptosis	In vivo (T2DM)	5% Saskatoon berry powder	4 weeks	Reduced the protein expression of CHOP	Zhao et al., 2015 [65]
Purple rice extract	Anti-inflammation, anti-fibrosis	In vivo (T1DM, DCM)	250 mg/kg/day	4 weeks	Downregulated COX-2, TLR4, and IL-6, decreased MMP-9 and TIMP-1 protein expression, reversed activation of p-IKKα/β, p-IκBα, and p-NF-κB proteins, reduced the activation of FGF2, p-ERL1/2, and uPA, as well as decreased TGF-β, p-MEK1/2, and CTGF	Chen et al., 2016 [66]
Black rice extract	Anti-apoptosis	In vivo (T1DM, DCM)	250 mg/kg/day	4 weeks	Limiting the protein levels of Fas ligand, Fas receptor, FADD, caspase-8, decreased Bad, Bak expression, and lessened cytochrome c accumulation, inhibited caspase-9, suppressed the increase in levels of cleaved caspase-3 and PARP expression	Huang et al., 2017 [67]
Commercial cyanidin-3-O-glucoside	Antioxidative	In vitro (palmitate induced insulin resistance)	20 μM	24 h	Increased activation of Nrf2, reversed P13K/Akt axis, attenuated eNOS expression, increased NO released, inhibited IRS-1 serine phosphorylation, restored IKKβ and JNK activation to normal levels	Fratantonio et al., 2017 [68]
Freeze-dried strawberry powder	Antioxidative, anti-inflammation	In vivo and in vitro (T2DM)	2.35% freeze-dried strawberry	10 weeks	Downregulated IκKβ, IκBα, NOX2, and NOX4 expression, reduced production of MCP-1/JE, KC, ICAM1, and VCAM1	Petersen et al., 2018 [69]
Commercial cyanidin-3-glucoside	Antioxidative, anti-inflammation, anti-apoptosis	In vivo (T2DM, DCM)	10 mg/kg/day	7 days	Increased SOD, decreased MDA, increased TNF-α and IL-6 levels, raised the level of Bcl-2, and reduced level of caspase-3 and BAX response	Li et al., 2018 [70]
Blueberry anthocyanin extract	Antioxidative	In vitro (High-glucose-induced HUVECs)	5 µg/mL	24 h	Decreased ROS generation, increased HO-1 and SOD expression, reduced NOX4 expression, enhanced NO and eNOS activation, increased P13K activity and breakdown of PKCzeta	Huang et al., 2020 [14]
Berry-derived anthocyanin	Antioxidative, anti-inflammation	In vitro	80 mg	12 weeks	Decreased H_2_O_2_-induced oxidative stress, inhibited the activation of NF-κB, reduced the levels of IL-6, reduced the activation of caspase-1 protein	Aboonabi et al., 2020 [71]
Anthocyanin-rich extract of sour cherry	Antioxidative, anti-inflammation	In vitro (high-glucose-induced HUVECs)	1 to 50 ng/µL	48 h and 7 days	Reduced ROS levels, downregulated TNF-α, IL-6, IL-8, and IL-1α expression, increased gene expression of NOS	Markovics et al., 2020 [5]
Blackcurrant	Anti-inflammation, anti-fibrosis	In vivo (T2DM)	200 mg/kg/day	10 weeks	Downregulated expression of elastin, collagen IV, IL-6, IL-1β, TNF-α, and TGF-β	Kim et al., 2021 [16]

## Data Availability

Data sharing not applicable.

## Abbreviations

ACE	Angiotensin-converting enzyme
AGE	Advanced glycation end product
CHOP	C/EBP homologous protein
COX	Cyclooxygenase
CTGF	Connective tissue growth factor
CVD	Cardiovascular disease
DCM	Diabetic cardiomyopathy
DM	Diabetes mellitus
ECM	Extracellular matrix
eNOS	Endothelial NO synthase
ERK	Extracellular signal-regulated kinase
FADD	Fas-associated death domain
HbA1c	Hemoglobin A1c
HDL	High-density lipoprotein
HO-1	Heme oxygenase-1
HUVEC	Human umbilical vein endothelial cell
ICAM-1	Intracellular adhesion molecule 1
IKKβ	Inhibitory kappa-B kinase β
IL	Interleukin
JNK	Jun N-terminal kinase
LDL	Low-density lipoprotein
MCP-1	Monocyte chemotactic protein 1
MDA	Malondialdehyde
MMP	Metalloproteinase
NF-κB	Nuclear factor kappa B
NLRP3	NLR family pyrin domain containing 3
NO	Nitric oxide
NOX	NADPH oxidase
Nrf2	Nuclear factor erythroid 2-related factor 2
PAI-1	Plasminogen activator inhibitor-1
PI3K	Phosphoinositide 3-kinase
PKC	Protein kinase C
PPARγ	Peroxisome proliferator-activated receptor-γ
ROS	Reactive oxygen species
SGLT1	Sodium-dependent glucose transporter 1
SOD	Superoxide dismutase
T1DM	Type 1 diabetes mellitus
T2DM	Type 2 diabetes mellitus
TGF-β	Transforming growth factor beta
TIMP-1	Tissue inhibitor of metalloproteinase-1
TLR4	Toll-like receptor 4
TNF-α	Tumor necrosis factor α
VCAM-1	Vascular adhesion molecule 1
XO-1	Xanthine oxidase-1
